# Hearing aids and recovery times: a study according to cognitive status

**DOI:** 10.5935/1808-8694.20130032

**Published:** 2015-11-02

**Authors:** Rosângela Ghiringhelli, Maria Cecilia Martinelli Iorio

**Affiliations:** a MSc Student at the Graduate Program in Human Communicaton Disorders - Speech and Hearing Department - Medical School of the University of São Paulo - UNIFESP/EPM; b Associate Professor at the Federal University of São Paulo (Associate Professor at the Federal University of São Paulo). Federal University of São Paulo - Department of Otorhinolaryngology and Human Communicaton Disorders - Hearing Disorders Course

**Keywords:** cognition, health of the elderly, hearing aids, questionnaires, speech perception

## Abstract

Studies have shown that elderly people with cognitive impairments benefit more from hearing aids with slower recovery times.

**Objective:**

To study participation constraints and speech recognition in noise of elderly subjects equipped with hearing aids of different recovery times according to cognitive impairment status.

**Method:**

Fifty subjects aged between 60 and 80 years were followed for four months. They were divided at first in groups of individuals without (G1; n = 24) and with (G2; n = 26) cognitive impairment based on results of the Alzheimer's Disease Assessment Scale - Cognitive Sub-scale test. Half the members of each group received hearing aids with faster recovery times and half got slower recovery aids, thus forming four groups: two without cognitive impairment (faster recovery - G1F; slower recovery - G1S) and two suspected for cognitive impairment (faster recovery - G2F; slower recovery - G2S). All subjects were interviewed, submitted to basic audiological assessment, asked to answer the Hearing Handicap Inventory for the Elderly questionnaire, and tested for speech recognition in noise. ANOVA, McNemar's test, and the Chi-square test were applied. The significance level was set at 5%.

**Results:**

There was significant improvement in participation constraint and speech recognition in noise with hearing aids alone. Sub-group G2F needed more favorable signal-to-noise ratios to recognize 50% of the speech in noise.

**Conclusion:**

Participation constraint and speech recognition in noise were improved regardless of recovery times or cognitive impairment status.

## INTRODUCTION

One of the most prevalent disorders among the aging population is presbycusis. Auditory function deterioration is one of the most severe impairments correlated to this process^1^, ^2^.

Elderly subjects with presbycusis have reduced auditory sensitivity and compromised speech intelligibility. The ensuing involvement of verbal communication skills may worsen overall quality of life^3^. This deterioration generates important sequelae, such as activity limitation and constrained participation in activities of daily living, leading to reduced socialization and, consequently, emotional alterations^4^.

Another alteration frequently associated with aging is the impairment of cognitive processes such as memory, attention, perception, comprehension, problem solving, language, and others. Research indicates the existence of a significant correlation between hearing loss and cognitive performance in the elderly^5^, ^6^, ^7^.

A study^8^ listed four possible explanations for the correlation between perception and cognition in aging subjects: cognitive decline as a symptom of diffuse neural degeneration; cognitive decline as a factor leading to perceptual decline; perceptual decline as a factor resulting in permanent cognitive decline; poor perceptual input as a factor leading to compromised cognitive performance.

These explanations motivated the development of research in an attempt to establish the link between hearing and cognition. Considering that perceptual decline and poor perceptual input may result in reduced cognitive performance, the author suggested audiological rehabilitation as a possible intervention.

Researchers from the Berlin Study Group on Aging concluded that the interaction between auditory and cognitive processing in elderly subjects with hearing loss results in increased effort to hear, thus limiting the use of the available mental processes for comprehension and recalling what was heard^8^. Individual differences and factors related to aging affect speech recognition in noise, but adjustments to the signal-to-noise ratio may minimize the difficulty inherent to this scenario.

The author^8^ reported that, in terms cognitive processes, audiological rehabilitation brings up two important implications. One of them is that, in addition to the age-related hearing impairment, the use of limited cognitive resources may constrain the participation of elderly subjects in activities of daily living which require hearing, comprehension, and communication. The other signifies that audibility may be partially or totally restored with the use of hearing aids, leading not only to improved speech perception but also to better cognitive performance in understanding and recalling what was heard.

In regards to hearing aid fitting, there is a known correlation between cognitive status and the benefit yielded by the hearing aids to various dynamic traits of comprehension. Release times are conventionally categorized as fast (< 100 ms, also known as “syllabic”) - in which the purpose is to maintain phoneme audibility - and slow (> 150 ms, called “automatic volume control”, or AVC) - in which the total volume of the signal is projected at a level comfortable for the hearing aid user^8^.

Recent studies have tested elderly individuals wearing syllabic and AVC hearing aids for speech recognition in noise. Subjects with better cognitive processing benefitted from fast release devices, whereas individuals with poorer cognitive skills did better with slow release hearing aids. Since then, device makers began to incorporate the outcomes of research into speech processing algorithms^8^, ^9^.

It is recommended that elderly subjects with presbycusis be referred to hearing aid fitting, once hearing loss is correlated to cognitive impairment and rehabilitation may be associated with overall cognitive improvement^10^.

Based on what has been stated, this study aimed to assess and compare the self-perceptions related to auditory constraints and performance at speech recognition in noise of elderly individuals before and after they were fitted with nonlinear hearing aids with different release times based on cognitive status.

## METHOD

The study was carried out in 2010 and 2011 after permit 0984/10 was issued by the institution's Research Ethics Committee. This longitudinal trial assessed and compared the performance of 50 elderly first-time users of nonlinear hearing aids aged between 60 and 80 years.

All enrolled individuals agreed to join the study and signed informed consent terms.

The following inclusion criteria were adopted in selecting the study sample: age equal to or greater than 60 and under 80 years; mild to moderately severe bilateral sensorineural hearing loss (mean hearing thresholds of 26 to 70 dB at 500, 1000, and 2000 Hz)^11^; not having used hearing aids before.

Participants were selected and placed in two groups, one featuring subjects without cognitive impairment (G1) and another comprised by individuals with cognitive impairment (G2). The Alzheimer's Disease Assessment Scale - Cognitive Sub-scale (ADAS-Cog) was used to form the groups. This scale has been translated and adapted into Brazilian Portuguese^12^ and applied by the author herself. The ADAS-Cog scale is made up of 11 items covering memory, language, praxis, and following commands. The top score in the cognitive sub-scale is 70 points. Higher scores mean more severe cognitive impairment. The level of education of the assessed subject must be considered before a test result indicative of cognitive impairment is rendered. Thus, cognitive impairment is diagnosed for scores above 23.3 for elderly subjects with up to four years of school education; for scores above 13.4 for elderly subjects with five to 11 years of schooling; and for scores above 11.1 for elderly subjects with 12 or more years of school education. Subjects with cognitive impairment were referred to specialized care after the completion of the tests.

Fifty subjects - 23 (46%) males and 27 (56%) females - were assigned to their respective groups, as follows: 24 individuals without cognitive impairment to group one (G1) and 26 with cognitive impairment to group two (G2).

The groups were further divided into two sub-groups: one with subjects fitted with nonlinear hearing aids with fast release time (320 ms) and the other with individuals fitted with slow release time devices (1280 ms). Participants were fitted with hearing aids of the same model and make.

The release time of the device was randomly assigned to the first patient on the group and repeated for every other subject in the list. Thus, four sub-groups were formed: G1F - 12 subjects without cognitive impairment fitted with fast release devices; G1S - 12 subjects without cognitive impairment fitted with slow release aids; G2F - 13 subjects with cognitive impairment fitted with fast release devices; G2S - 13 subjects with cognitive impairment fitted with slow release devices.

The subjects were advised and followed up for four months after having the devices fitted, and were then reassessed. All enrolled group members were first submitted to an interview and basic audiological evaluation, followed by a research protocol that included the Hearing Handicap Inventory for the Elderly (HHIE) self-assessment tool and the *Listas de Sentenças em Português - LSP* (Brazilian Portuguese Sentence List) speech recognition test.

The HHIE^13^ was used to assess participation constraints in activities of daily living. This scale includes 25 questions divided into sub-scales Social/Situational (S) and Emotional (E)^14^. Points are given to each of the three possible answers: “yes” = 4 points, “sometimes” = 2 points, and “no” = no points. The global score may range between zero and 100% (full perception of participation constraint). Higher scores mean greater perception by the individual of his/her participation constraint^15^: 0% to 16% - no self-perceived limitation; 18% to 42% - mild to moderate self-perception of constraint; and above 42% - severe/significant self-perception of limitation. The scale was applied by the author in a silent room in the form of an interview.

The LSP^16^ was used to analyze the signal-to-noise ratio. The LSP contains a list of 25 sentences in Brazilian Portuguese (list 1A) and seven lists with ten sentences each, and competitive noise within the range of speech.

The evaluations were carried out in a soundproof room, with the subjects wearing hearing aids fitted in accordance with the data taken from their individual audiometric tests and positioned one meter away from the sound source placed on 0° azimuth, i.e., in front of the subjects. In order to obtain free field sound pressure levels, measurements were made as per the procedure described in previous studies^17^.

The application of this tool was based on the ascending-descending strategy^18^, which allows the determination of the thresholds of speech recognition in noise (S/R ratio) at which subjects can accurately recognize 50% of the presented sentences. The signal-to-noise ratio is the difference between the mean level of the sentences and the level of competing noise. The ratio is negative when the subject is able to recognize speech at a level lower than the level of the noise.

Chart 1 shows the parameters used in the administration of the LSP test.

**Chart 1:** Parameters for LSP administration.

Speech stimulus level	Variable speech stimulus levels
List of sentences used for practice	1A
List of sentences: subject without hearing aids	1B
List of sentences: subject with hearing aids	2B
List of sentences in reassessment	3B
Initial level of speech stimulus	65 dB (A)
Noise level (constant)	65 dB (A)
Increment for first sentences	4 dB
Increment of sentences from mistake	2 dB
Device maximum output	120 dB

### Statistical analysis

HHIE scores and signal-to-noise ratios were analyzed using descriptive statistics. Analysis of va-riance^19^ was used to compare the mean scores before and after fitting. The mean differences seen between tests were calculated using a 95% confidence interval. A statistical significance level of 0.05 was used, and significant p-values were highlighted with an asterisk (*).

## RESULTS

**Figure 1:**
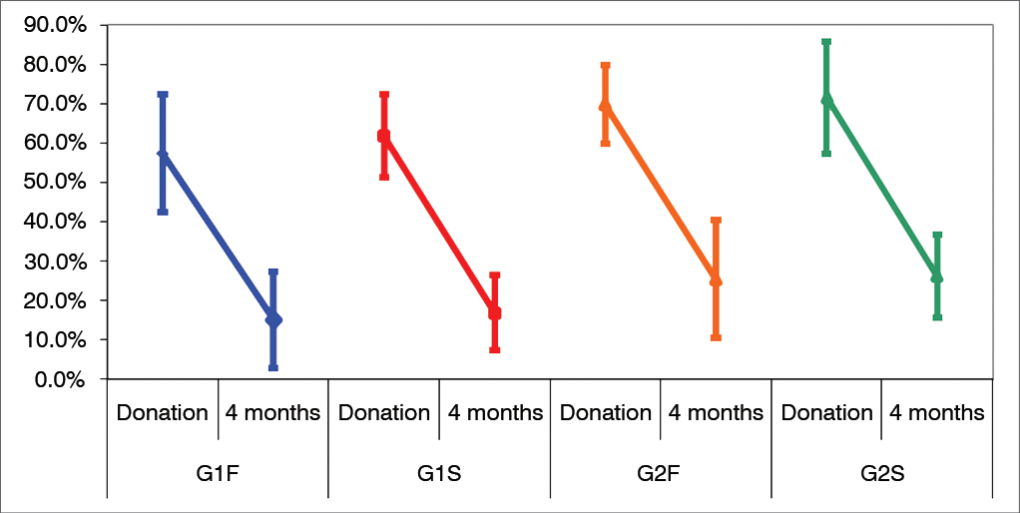
Differences between the pre and post-fitting stages in the HHIE scale. Confidence interval for the mean value: mean ± 1.96 * standard deviation/ $\sqrt{\left(\text{n}-1\right)}$. G1F: Sub-group of subjects without cognitive impairment and fast release time devices; G1S: Sub-group of subjects without cognitive impairment and slow release time devices; G2F: Sub-group of subjects with cognitive impairment and fast release time devices; G2S: Sub-group of subjects with cognitive impairment and slow release time devices.

Tables 1 and 2 and Figure 1 show the analysis of the HHIE scores of the four groups at two different times (before and after device fitting). Two-way analysis of variation (ANOVA) (group and stage) was used to that end. Table 2 shows the ANOVA results.

**Table 1:** Descriptive statistics for HHIE scale, before and after intervention, of the four sub-groups.

	Group							
	G1F		G1S		G2F		G2S	
	Before	After	Before	After	Before	After	Before	After
Mean	57.33%	15.00%	61.83%	16.83%	69.69%	25.38%	71.38%	26.15%
SD	25.22%	20.74%	17.84%	16.12%	17.55%	26.41%	25.24%	18.47%
n	12	12	12	12	13	13	13	13

G1F: Sub-group of subjects without cognitive impairment and fast release time devices; G1S: Sub-group of subjects without cognitive impairment and slow release time devices; G2F: Sub-group of subjects with cognitive impairment and fast release time devices; G2S: Sub-group of subjects with cognitive impairment and slow release time devices; SD: Standard deviation; n: number of subjects.

**Table 2:** ANOVA results for the HHIE scale.

Effects	*p*-value
Group	0.2227
Stage	< 0.0001*
Group vs. Stage	0.9904

Significant differences were observed in the HHIE scores before and after nonlinear hearing aid fitting in all sub-groups four months into wearing the devices. No significant differences were seen between groups.

**Figure 2:**
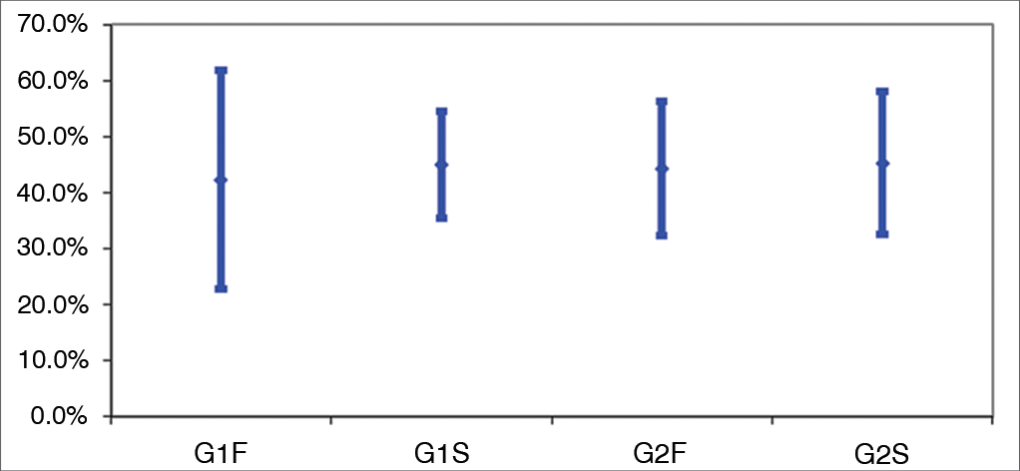
Analysis of benefit among groups in the HHIE scale. Confidence interval for the mean value: mean ± 1.96 * standard deviation/ $\sqrt{\left(\text{n}-1\right)}$. G1F: Sub-group of subjects without cognitive impairment and fast release time devices; G1S: Sub-group of subjects without cognitive impairment and slow release time devices; G2F: Sub-group of subjects with cognitive impairment and fast release time devices; G2S: Sub-group of subjects with cognitive impairment and slow release time devices.

The benefit analysis done using HHIE in the four groups was carried out through one-way ANOVA. Results are shown on Table 3 and depicted on Figure 2.

**Table 3:** Mean values and standard deviations for HHIE benefit analysis.

	Group			
	G1F	G1S	G2F	G2S
Mean	42.33%	45.00%	44.31%	45.23%
Standard deviation	33.02%	16.21%	21.10%	22.55%
n	12	12	13	13

G1F: Sub-group of subjects without cognitive impairment and fast release time devices; G1S: Sub-group of subjects without cognitive impairment and slow release time devices; G2F: Sub-group of subjects with cognitive impairment and fast release time devices; G2S: Sub-group of subjects with cognitive impairment and slow release time devices; n: number of subjects; ** ANOVA (*p*) = 0.990.

Table 4 shows the analysis of the LSP test applied to the four sub-groups before and after hearing aid fitting. Two-way ANOVA (group and stage) was used in this case. Table 5 shows the ANOVA results.

**Table 4:** Mean values and standard deviations for the four sub-groups before and after the ftting of hearing aids.

	Group							
	G1F		G1S		G2F		G2S	
	Before	After	Before	After	Before	After	Before	After
Mean	4.456	2.611	5.041	3.000	8.068	5.603	4.811	3.212
Standard deviation	3.7716	2.7999	4.6764	3.4266	4.5389	4.1261	3.4959	2.7018
n	12	12	12	12	13	13	13	13

G1F: Sub-group of subjects without cognitive impairment and fast release time devices; G1S: Sub-group of subjects without cognitive impairment and slow release time devices; G2F: Sub-group of subjects with cognitive impairment and fast release time devices; G2S: Sub-group of subjects with cognitive impairment and slow release time devices; n: number of subjects in group.

**Table 5:** ANOVA results for the LSP test.

Effects	*p*-value
Group	0.0529
Stages	0.0006*
Group vs. Stage	0.9474

**Figure 3:**
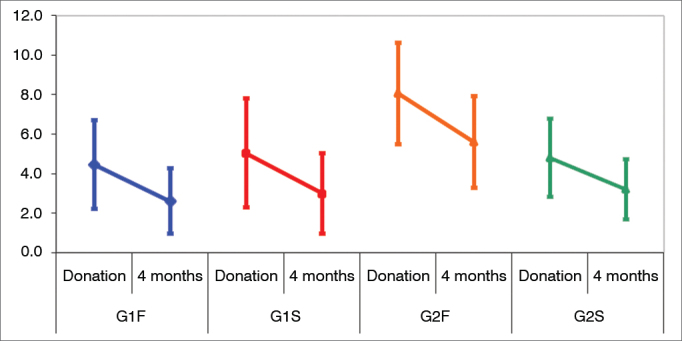
Differences between groups in the LSP test. Confdence interval for the mean value: mean ± 1.96 * standard deviation/ $\sqrt{\left(\text{n}-1\right)}$. G1F: Sub-group of subjects without cognitive impairment and fast release time devices; G1S: Sub-group of subjects without cognitive impairment and slow release time devices; G2F: Sub-group of subjects with cognitive impairment and fast release time devices; G2S: Sub-group of subjects with cognitive impairment and slow release time devices.

Significant differences were seen between stages, but not between groups or interaction. The differences between groups were nearly significant (p ranging between 0.05 and 0.10). The possible differences were searched through multiple post-hoc comparisons shown on Table 6. Figure 3 illustrates the differences.

**Table 6:** POST HOC for effect Group (LSP test).

Frequency	G1F	G1S	G2F	G2S
G1F	-	-	-	-
G1S	0.7146	-	-	-
G2F	0.0144*	0.0354*	-	-
G2S	0.7144	0.9944	0.0315*	-

G1F: Sub-group of subjects without cognitive impairment and fast release time devices; G1S: Sub-group of subjects without cognitive impairment and slow release time devices; G2F: Sub-group of subjects with cognitive impairment and fast release time devices; G2S: Sub-group of subjects with cognitive impairment and slow release time devices; SD: Standard deviation.

Significant difference was seen between stages for all sub-groups as subject scores in the LSP were lower into four months of wearing nonlinear hearing aids.

Group G2F (cognitive impairment and fast release devices) had higher scores than the other groups.

## DISCUSSION

Hearing loss introduces auditory and non-auditory impacts, such as isolation from society and family.

The purpose of hearing aids is to improve subject audibility and include users in society. It is important to look into the auditory constraints imposed by hearing loss before and after subjects are fitted with hearing aids, as this analysis captures relevant clinical information that would otherwise be missed in objective assessments. The HHIE is frequently used to that end.

The mean HHIE scores attained before and after hearing aid fitting were 59.6% and 15.9% for G1 subjects (without cognitive impairment) and 70.5% and 25.8% for G2 members (suspected cognitive impairment) respectively, with a statistically significant^20^ *p* < 0.001. These results were consistent with the perceived existence of severe participation constraints before the subjects of both groups were fitted with hearing aids, the elimination of such perception after the hearing aids were fitted in G1 subjects, and the still remaining mild perception of limitation reported by individuals in G2.

When the four groups were considered it was noted that the individuals suspected for cognitive disorders had higher scores than the subjects on the group without impairment before and after undergoing treatment with a speech and hearing therapist. Differently from other groups, they failed to reach scores consistent with absence of perceived participation constraints after being fitted with hearing aids. This situation revealed that these patients experienced more limitations in participating in activities of daily living. When the benefit of hearing aids was considered, it was found that all groups had a reduction of 42% to 45% in participation constraints.

According to the literature, a reduction of 19% in perceived participation constraints after the fitting of hearing aids amounts to a significant improvement when the HHIE scale is applied in the form of an interview. When the HHIE is answered by the patients without the aid of an interviewer, reductions of 36% or more amount to significant improvement^20^.

Other authors^21^ have described that elderly men report higher degrees of perceived limitations and greater benefits after intervention by a speech and hearing therapist.

In order to communicate well in situations of conversation, individuals must decode and identify the incoming message by integrating auditory and language processing. However, if the decoding function is compromised by poor performance of the peripheral auditory system, other cognitive resources have to be mustered so that good communication can occur^22^. When greater effort is made to hear and more cognitive resources are used in the comprehension of basic sounds, memory and cognition suffer while processing discourse. Hearing aids can be a valuable addition in this circumstance.

Studies have shown that the auditory stimulation provided by the amplification of sounds leads to the reorganization of the auditory pathways, and possibly to improvements in the reception and organization of sound stimuli. Consequently, one's speech recognition skills improve gradually^23^, ^24^. This study found supporting evidences by using hearing aids as a form of auditory rehabilitation unaccompanied by other procedures such as formal auditory training.

The findings in this study were also in agreement with the research of other authors^25^, and supported the use of hearing aids and the benefits they yield to the social and personal lives of elderly subjects with presbycusis, regardless of their individual cognitive status, by reducing self-perception of participation constraints in activities of daily living.

This study also noted the importance of using hearing aids to improve the cognitive functions (memory and attention) of elderly individuals with hearing loss, regardless of hearing aid release time.

The ADAS-Cog test was used in this study with the purpose of dividing subjects in two groups - without cognitive impairment (G1) and suspected for cognitive impairment (G2) - and to analyze the impact of hearing loss upon cognitive function. After the subjects were given hearing aids, improvements in auditory perception, communication, socialization, and cognition (memory and attention) were reported by individuals from both groups. Therefore, the use of hearing aids by elderly subjects may prevent the occurrence of cognitive alterations associated with auditory perception and attention, and consequently improve the overall quality of life of the elderly. Release times did not affect the results attained by the groups.

The reasons for the significant correlation found between cognitive status and fast release devices are yet to be clarified. Temporal auditory processing or some other factor such as cerebral plasticity may mediate this correlation^8^.

Situations of everyday life are normally associated with the presence of competing noise. Individuals with normal hearing experience difficulties hearing and recognizing speech in these conditions. Thus, it is important to assess the elderly in communication situations that resemble reality with tests to assess speech recognition in noise. The test chosen in this study was the LSP^16^.

This study showed a reduction on the group scores after hearing aid fitting, a finding correlated with better performance in situations of speech in noise.

Speech recognition is one of the biggest challenges for elderly subjects with hearing loss, particularly when competing noises are present. The signal-to-noise ratio is calculated as the difference between the intensity of one signal (speech) and the competing noise presented simultaneously. Higher S/N ratios indicate more difficulty understanding speech in noise, both for people with normal hearing and subjects with hearing loss, the elderly in particular.

Other authors^26^ have concluded that older individuals with fast and slow release time devices did not perform as well as younger adults in speech recognition in noise tests, and that no differences were seen among users of fast and slow release time hearing aids. There was no interaction between age, degree of hearing loss, and release time. Based on these results, the fitting of hearing aids should not consider solely the age of the patient.

The minimum values seen in this study for S/N ratio varied by 1.1 dB on Group 1 (no cognitive impairment) and 1.6 dB on Group 2 (suspected cognitive impairment). Studies have shown that a 1 dB change in the S/N ratio may lead to variations in speech recognition of up to 18%^27^, a significant difference for elderly subjects with and without cognitive impairment.

The minimum values seen in this study for S/N ratio before and after the fitting of hearing aids (-1.7 and -2.8 for Group 1; and 0 and -1.6 for Group 2 respectively) are in agreement with other studies^28^ in which the mean S/N ratio was -2.37 for nonlinear hearing aid users, with a minimum value of -4.13.

Patients in the G2F group had a higher S/N ratio to recognize 50% of the sentences before and after intervention by a speech and hearing therapist only by being fitted with hearing aids. Other factors must be analyzed to justify these findings, such as time of sensory deprivation.

The findings in this study indicated reduced participation constraints four months after the subjects started using nonlinear hearing aids. The same was seen for speech recognition in noise.

## CONCLUSION

Patients using hearing aids for four months improved from participation constraints and performed better in speech recognition in noise tests regardless of their cognitive status and the release time setting of their hearing aids.
